# Effects of alfa-hydroxy-isocaproic acid on body composition, DOMS and performance in athletes

**DOI:** 10.1186/1550-2783-7-1

**Published:** 2010-01-05

**Authors:** Antti A Mero, Tuomo Ojala, Juha J Hulmi, Risto Puurtinen, Tuomo AM Karila, Timo Seppälä

**Affiliations:** 1 Department of Biology of Physical Activity, University of Jyväskylä, Jyväskylä, Finland; 2 Hospital Orton, Helsinki, Finland; 3 Orthopaedic Department, Gisborne Hospital, New Zealand; 4 Department of Alcohol, Drugs and Addiction, National Institute for Health and Welfare, Helsinki, Finland

## Abstract

**Background:**

Alfa-Hydroxy-isocaproic acid (HICA) is an end product of leucine metabolism in human tissues such as muscle and connective tissue. According to the clinical and experimental studies, HICA can be considered as an anti-catabolic substance. The present study investigated the effects of HICA supplementation on body composition, delayed onset of muscle soreness (DOMS) and physical performance of athletes during a training period.

**Methods:**

Fifteen healthy male soccer players (age 22.1+/-3.9 yr) volunteered for the 4-week double-blind study during an intensive training period. The subjects in the group HICA (n = 8) received 583 mg of sodium salt of HICA (corresponding 500 mg of HICA) mixed with liquid three times a day for 4 weeks, and those in the group PLACEBO (n = 7) received 650 mg of maltodextrin mixed with liquid three times a day for the same period. According to a weekly training schedule, they practiced soccer 3 - 4 times a week, had strength training 1 - 2 times a week, and had one soccer game during the study. The subjects were required to keep diaries on training, nutrition, and symptoms of DOMS. Body composition was evaluated with a dual-energy X-ray absorptiometry (DXA) before and after the 4-week period. Muscle strength and running velocity were measured with field tests.

**Results:**

As compared to placebo, the HICA supplementation increased significantly body weight (p < 0.005) and whole lean body mass (p < 0.05) while fat mass remained constant. The lean body mass of lower extremities increased by 400 g in HICA but decreased by 150 g in PLACEBO during the study. This difference between the groups was significant (p < 0.01). The HICA supplementation decreased the whole body DOMS symptoms in the 4^th ^week of the treatment (p < 0.05) when compared to placebo. Muscle strength and running velocity did not differ between the groups.

**Conclusion:**

Already a 4-week HICA supplementation of 1.5 g a day leads to small increases in muscle mass during an intensive training period in soccer athletes.

## Background

DL-α-hydroxy-isocaproic acid (HICA), also known as leucic acid or DL-2-hydroxy-4-methylvaleric acid, is an end product of leucine metabolism in human tissues such as muscle and connective tissue [1,2]. Some foodstuffs produced by fermentation, e.g. certain cheeses, wines and soy sauce contain HICA [3-7]. According to the clinical and experimental studies HICA can be considered as an anti-catabolic substance [8-15]. There is an evidence of a direct in vitro inhibitory effect of HICA on various matrix metalloproteinase enzymes, which are responsible for degradation of various connective and protein tissues [14]. The delayed onset of muscle soreness (DOMS) is the sensation of muscular discomfort and pain during active contractions that occurs in a delayed fashion after strenuous exercise. Subjects with DOMS have painful, tender, and swollen muscles with reduced range of motion of adjacent joints especially after unaccustomed exercise [16,17]. In addition to muscle tenderness with palpation, prolonged strength loss and a reduced range of motion are observed. These symptoms develop 24 to 48 hours after exercise, and they disappear within 5 to 7 days [16,17]. The pathophysiology of DOMS remains still undetermined, but it has been reported that after strenuous exercise muscle cell damage and inflammatory cells are observed in damaged muscle [16,17].

Although leucine has a unique role as a promoter of protein synthesis [18], maybe especially the metabolites of leucine decrease breakdown of proteins, particularly muscle proteins [11]. The roles and mechanisms of actions of leucine and its metabolites are not clear and even confusing. For instance, α-ketoisocaproate (KIC), derived from leucine by transamination, is anti-catabolic and reduces muscle protein degradation when given as intravenous infusion [11]. On the other hand, it is a potent inhibitor of branched-chain α-keto acid dehydrogenase kinase and may lead to increased catabolism of branched chain amino acids (BCAAs) [19]. β-Hydroxy β-methylbutyric acid (HMB) or β-hydroxy β-methylbutyrate is another metabolite of leucine and plays also a role in protein synthesis and breakdown [20]. Recently [21], it was observed that 14 of HMB and KIC supplementation reduced signs and symptoms of exercise-induced muscle damage in non-resistance trained males following a single bout of resistance exercise emphasizing eccentric contractions.

There are separate mechanisms to control protein synthesis and proteolysis [22]. Tischler et al [11] suggested that the first step in controlling muscle proteolysis by leucine is the oxidation of leucine, catalyzed by aminotransferase enzyme. The end product of the reaction is keto leucine (α-ketoisocaproate, KIC) but, in certain situations, it can be HICA as well. It is suggested that the aminotransferase enzyme is responsible to oxidize leucine both to its keto (KIC) and to its hydroxyl form (HICA) and both reactions are reversible [23]. The reaction between keto and hydroxyl leucine is an equilibrium reaction with oxidoreduction equilibrium constant (thermodynamic constant) Keq = 3.1 ± 0.2 × 10-12 mol/l and the reaction half time is 230 min towards oxygenation in human. Keto acid is irreversibly oxidized by mitochondrial ketoacid dehydrogenaze [24]. Irreversible degradation of keto acids is higher in liver than that in muscle [24]. The branched-chain α-keto acid dehydrogenase complex is the most important regulatory enzyme in the catabolism of leucine [25]. The skeletal muscle is considered to be the initial site of BCAA catabolism because of its high activity of BCAA aminotransferase [2].

In our open pilot study with wrestlers [15; unpublished] we assessed the effects of HICA on body composition and exercise induced DOMS. National top wrestlers (n = 7, 79.7 ± 4.5 kg, 26 ± 6 yrs) took 0.496 g of HICA three times per day after intensive training sessions for 42 days. They had at least 10 training sessions a week, each lasting from 1.5 to 2.5 hours. Since the subjects were competitive athletes they had records on their weights for years during their competition careers. During six weeks before the HICA period there were no essential changes in their weights. At least for the 6-week period before and during the 42-day trial daily diets and the number, intensity, and duration of daily training sessions of wrestlers were kept constant. According to DXA measurements the mean body weight gain during the treatment period was 0.84 ± 1.0 kg (± SD). Bone mass was not changed but total lean soft tissue mass was increased statically significantly. The most important finding of the pilot study was, however, that subjects when using HICA did not suffer from DOMS symptoms at all or they suffered markedly less than before the treatment with HICA. No changes in blood pressure, heart rate or laboratory blood values were associated with the use of HICA suggesting that its use is safe.

Consequently, the aim of this study was to investigate the effects of HICA supplementation on body composition, DOMS symptoms and physical performance during a controlled one month training period in soccer players. Our hypothesis was that HICA would increase total lean soft tissue mass, would decrease DOMS symptoms and would improve physical performance during training.

## Methods

### Subjects

The subjects were fifteen healthy male soccer players (age 22.1 ± 3.9 yr) in the local club. They signed a written consent which was approved by the local University Ethics Committee.

### Study design

This study was a double-blind, randomized, placebo controlled experiment. At the beginning of study the subjects were randomized to two groups: group HICA; n = 8, age 22.8 ± 6.4 yr, height 178.9 ± 6.8 cm, body fat 14.1 ± 3.9% and group PLACEBO; n = 7; age 21.3 ± 2.3 yr, height 178.4 ± 5.1 cm, body fat 12.5 ± 3.0%; mean ± SD. There were no differences in baseline parameters between the groups. The loading period with HICA or PLACEBO lasted four weeks and the similar tests were performed before and after the loading period. The subjects were familiarized with the tests well because similar tests were used in their normal training.

#### Loading

The subjects in the HICA group ingested DL-α-hydroxy-isocaproic acid (alfaHICA™ Elmomed Ltd, Helsinki, Finland) and the subjects in the PLACEBO group received maltodextrin (Manninen Nutraceuticals Ltd, Oulu, Finland). Both supplements were powders and three doses each were ingested per day. One HICA dose was 583 mg as sodium salt (corresponding 500 mg of HICA) mixed with juice or water. One PLACEBO dose included 650 mg maltodextrin mixed also with juice or water. Both powders were scaled and packed ready for the subjects in 1.5 ml Eppendorf tubes. The supplements were advised to ingest three times per day in equal time intervals with meals.

#### Training

Training consisted of 5-7 training sessions per week including 3-4 soccer sessions, 1-2 resistance exercise sessions, and one match. Resistance exercise session included both maximal strength and speed-strength exercises. All subjects were advised to keep training diaries on which they marked all training exercises as well as subjective evaluation of training alertness and the morning onset of delayed muscle soreness (DOMS) in lower and upper extremities. In both assessments the scale was from 1 to 5 where 5 is the best training alertness and the strongest soreness in the muscles. It has been shown earlier that a correlation coefficient between repeated measurements of muscle soreness is good (r = 0.96; [26]). Each subject was individually supervised how to keep training diaries and to report DOMS.

#### Nutrition

Before the beginning of the study, each subject was supervised to continue his normal sport nutrition program. On the testing day the subjects were supervised not to use any sport or dietary supplements. They were supervised also to keep food diaries for five days in the 4-week period for what they were provided with specific verbal and written instructions and procedures for reporting detailed dietary intake, including how to record portions by using household measures, exact brand names and preparation techniques. Dietary intake of the subjects was registered for five days including Saturday and Sunday. The food diaries were analyzed using the Micro Nutrica nutrient-analysis software (version 3.11, Social Insurance Institution of Finland).

## Data collection and analysis

**Figure 1:**
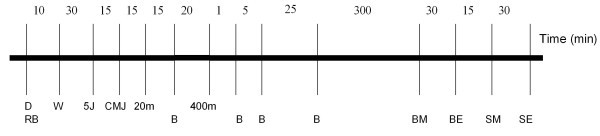
**Test protocol before and after the 4-week loading period**. D = DXA, RB = rest blood sample, W = standard warm up, 5J = standing 5-jump, CMJ = counter movement jump, 20 m = 20 m sprint, B = blood sample, 400 m = 400 m run, BM = bench 1RM, BE = bench strength endurance, SM = squat 1RM, SE = squat strength endurance.

Each subject was tested before and after the 4-week (28 days) loading period at the same time of day (Figure 1).

### Blood sampling

In the morning blood samples were taken from an antecubital vein in the sitting position. Two milliliters blood from a vein was taken in K2 EDTA tubes (Terumo Medical Co., Leuven, Belgium) for measurements of hemoglobin and hematocrit concentration with a Sysmex KX 21N Analyzer (Sysmex Co., Kobe, Japan). The intra-assay coefficient of variation (CV) was 1.5% for hemoglobin and 2.0% for hematocrit. After the 400 m running test (immediately, 5 min and 30 min) five milliliters of blood was taken in lithium-heparin tubes (Terumo Medical Co., Leuven, Belgium) for measurement of lactate (Biosen C line, Sport; EKF Magdeburg, Germany) and pH with a Nova Biomedical STAT Profile PhOX Plus L Analyzer (Nova Biomedical, Waltham, MA, USA). The intra-assay CV was 3.0% for lactate and 0.1% for pH.

### Body composition

Total body composition changes were determined using a dual-energy X-ray absorptiometry device (DXA; Lunar Prodigy Densitometer, GE Lunar Corporation, Madison, WI, USA). This method can differentiate total body bone mineral density (BMD), total percentage fat, total body tissue mass, fat mass, lean mass, bone mineral content (BMC), and total bone calcium with CVs of 0.62, 1.89, 0.63, 2.0, 1.11, 1.10, and 1.09%, respectively [27]

### Jumping ability

Maximal standing 5-jump was used to measure explosiveness of leg extensor muscles in horizontal direction [28]. Maximal vertical jumping ability was measured using a counter movement jump (CMJ) on a contact mat with a clock [29]. In both indoor tests the best performance of three trials (recovery from 3 to 5 minutes between the trials) was selected for the final analysis.

### Running tests

Both 20 m and 400 m run were performed indoors. Acceleration running speed was measured with a standing start over 20 m. The subject was standing 0.7 m from the first photocell gate and then accelerated maximally over 20 m to the second photocell gate (accuracy of 0.01s in time measurement). The fastest run of three trials (recovery 5 minutes) was selected to the final analysis. The indoor track was 200 m on which each subject ran alone maximally 400 m. Running times were recorded with stopwatches by two experienced investigators, and a mean performance time (accuracy of 0.1s) was calculated for the analysis. Subjects were instructed and verbally encouraged to give a maximal effort for the performance.

### Strength tests

Maximum strength (1RM) was measured in bench press with a free barbell and in full squat using a Smith machine. Strength endurance was measured performing as many repetitions as possible using a 50% load of 1RM in both bench press and in full squat. The test order was as follows: bench press 1RM, bench press strength endurance, full squat 1RM, and full squat strength endurance. Recoveries between trials were from three to five minutes in each test and at least five minutes between different tests. Continuous verbal encouragement was given during all the test performances.

### Statistical Analyses

The Analysis of Variance (A Group-by-Time Factorial ANOVA) was used to assess statistical differences between the treatment groups. Data were handled as changes between the measurements before and after the treatments. Further, bonferroni corrected paired t-test was used to compare values before and after treatments. P ≤ 0.05 was regarded as statistically significant. Statistical analyses were carried out using the software program Systat for Windows (Statistics, Version 9, Evanston, IL, USA, 1992). The results are presented as means ± SD.

## Results

### Training and Nutrition

There were no differences in training between the groups of HICA and PLACEBO during the 4-week study period. The training amount across the study period consisted of 13 ± 3 soccer sessions, 4 ± 1 strength training sessions and 3 ± 1 matches.

The subjects ate similarly in both groups and the average daily macronutrient intake during five days across the 4-week study period was as follows: energy 11183 ± 2361 kJ, protein 119 ± 37 g, carbohydrate 341 ± 87 g, and fat 82 ± 23 g.

### Hemoglobin and hematocrit

There were no differences in hemoglobin or hematocrit between the groups of HICA and PLACEBO or between before and after measurements in each group. The average value for the total subject group was 150 ± 6.4 g/l in hemoglobin and 44 ± 0.03 in hematocrit.

### Body composition

**Figure 2:**
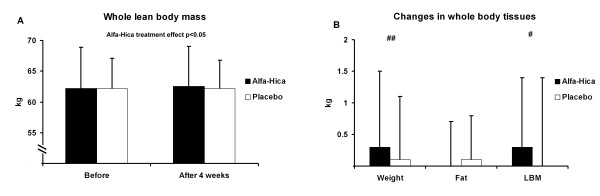
**Whole lean body mass (A) and changes in whole body tissues (B)**. Data are mean ± SD. ## represents (p < 0.005) and # represents (p < 0.05) difference in change between before and after measurement between the groups.

**Figure 3:**
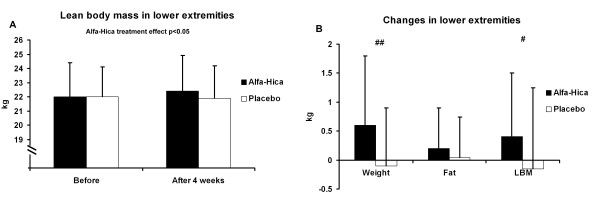
**Lean body mass of lower extremities (A) and the changes of its components in lower extremities (B)**. Data are mean ± SD. ## represents (p < 0.001) and # represents (p < 0.01) difference in change between before and after measurement between the groups

**Figure 4:**
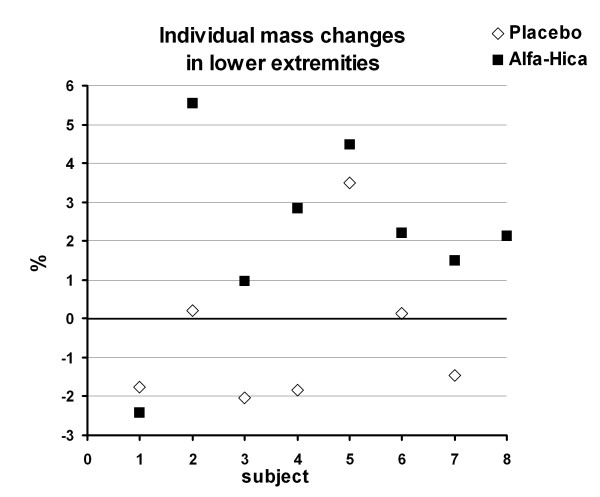
**Individual relative LBM changes in lower extremities**. Significance between groups p < 0.05

Body weight was in the HICA group before and after the 4-week study period 72.6 ± 9.1 kg and 72.9 ± 8.6 kg and in the PLACEBO group 70.0 ± 5.2 kg and 70.1 ± 5.1 kg, respectively. The difference between the treatments was significant in body weight (p < 0.005), in whole lean body mass (LBM: before 62.2 ± 6.7 and after 62.5 ± 6.5 for HICA and before 62.2 ± 4.9 and after 62.2 ± 4.6 for PLACEBO; p < 0.05; Figure 2A) while fat mass remained constant. Also bone mass (3.6 ± 0.1 kg) remained constant in both groups. The absolute changes were significant in weight (p < 0.005) and in LBM (p < 0.05) (Figure 2B). The difference between the treatments was significant also in lean body mass of lower extremities (p < 0.05) (Figure 3A). The lean body mass of lower extremities increased by 400 g in the HICA group but decreased by 150 g in the PLACEBO group and the changes between the groups differed significantly (p < 0.01) (Figure 3B). Individual changes in relative LBM of lower extremities are presented in Figure 4. There were no differences between the groups in body composition of upper extremities.

### Performance

The performance variables are presented in Table 1. There were no significant differences between the groups HICA and PLACEBO in any of the variables.

**Table 1:** Physical performance before and after the 4-week period

Variable	HICA		PLACEBO	
	Before	After	Before	After
5-jump (m)	13.44 ± 0.71	13.80 ± 0.73	13.22 ± 0.70	13.63 ± 0.91
CMJ (m)	43.5 ± 0.03	42.8 ± 0.06	42.3 ± 0.06	44.2 ± 0.05
20 m (s)	3.02 ± 0.06	3.04 ± 0.11	2.96 ± 0.05	2.98 ± 0.06
400 m (s)	61.3 ± 1.8	61.7 ± 1.6	60.2 ± 1.7	59.1 ± 0.7
pH ^a^	7.09 ± 0.11	7.17 ± 0.12	7.12 ± 0.12	7.17 ± 0.12
lactate (mmol/l) ^a^	13.6 ± 1.3	14.5 ± 2.2	14.4 ± 2.8	14.2 ± 2.9
Bench press 1RM (kg)	87.5 ± 21.0	87.9 ± 20.9	82.5 ± 13.5	83.3 ± 14.6
Strength endurance (reps)	31 ± 3	32 ± 4	28 ± 2	31 ± 3
Full squat 1RM (kg)	120 ± 19	130 ± 24	131 ± 29	138 ± 16
Strength endurance (reps)	31 ± 8	47 ± 5	36 ± 10	38 ± 11

Data are means ± SDs. ^a ^pH is the lowest value and lactate is the highest value after 400 m

### DOMS and training alertness

The HICA supplementation decreased significantly (p < 0.05) the whole body DOMS symptoms only in the 4^th ^week of the treatment (1.4 ± 0.3) when compared to placebo (1.8 ± 0.2) (all weeks 1.5 ± 0.3 for HICA and 1.7 ± 0.4 for PLACEBO; mean ± SD). Training alertness was during every study week slightly better in the HICA group (3.6 ± 0.5; 4.2 ± 0.5; 4.1 ± 0.5; 4.3 ± 0.6) compared to the PLACEBO group (3.3 ± 0.6; 3.0 ± 0.9; 3.4 ± 1.1; 3.4 ± 0.8) but significantly (p < 0.05) better only in the second week.

## Discussion

### Main results

The 4-week supplementation with HICA increased the whole lean body mass of the soccer players. This increase (400 g) was emphasized in lower extremities. Also the subjects in the HICA group felt milder DOMS compared to the subjects in the PLACEBO group. There were no differences between the groups in any of the performance variables.

### Body composition

The main result of this study was that lean body mass increased with HICA during the 4-week training period. Consequently, it is probable that skeletal muscle mass has increased especially in the lower extremities of the soccer players, because the main training and playing is leg work. The precision of DXA for lean body mass is 1.11% as we mentioned in the methods. The result in lower extremity change was small - in the HICA group there was a mean increase of 400 g (~2%) and in PLACEBO a decrease of 150 g (< 1%). Taking into account this short duration of the experiment period the difference between the groups can be considered rather clear (550 g). Looking also at the individual mass changes we can see a clear difference between the groups. Only one subject from each group is within another group. Human skeletal muscle protein metabolism has received significant attention over the past few decades because of its relevance to sport, physical inactivity, aging, and disease processes [30]. The importance of skeletal muscle is obvious since it comprises about 40% of body weight, constitutes between 50 and 75% of all proteins [31], and is important for locomotion. However, it is also important as an amino acid reservoir, for energy consumption and for fuels for other tissues (e.g., brain, immune cells). Skeletal muscle proteins have regular turnover such that 1 - 2% of proteins are synthesized and broken down daily [32]. The turnover of proteins involves the ongoing processes of protein synthesis and breakdown. A positive net protein balance occurs when proteins accumulate in excess of their removal (e.g., following nutrient ingestion), whereas a negative net protein balance occurs when the breakdown of proteins exceeds that of their synthesis (e.g., fasting). Indeed, protein, essential amino acids (particularly leucine) and resistance exercise but also endurance exercise [33] are powerful stimulators of skeletal muscle protein synthesis in animal and human models [34-37] and eventually skeletal muscle hypertrophy [18].

DL-α-hydroxy-isocaproic acid (HICA) is a physiological agent which is normally present in the human body in small amounts. Plasma concentration of HICA in healthy adults is 0.25 ± 0.02 mmol/l, that of its correspondent keto acid is 21.6 ± 2.1 mmol/l, and in circulation HICA is not bound to plasma proteins [1]. It can be measured from human plasma, urine and amniotic fluid as well [38-40]. It has been earlier [41] speculated that leucine alone accounts for about 60% of the total effectiveness of the group of the regulatory amino acids (leucine, tyrosine, glutamine, proline, methione, histidine, and tryptophan) to inhibit the deprivation-induced protein degradation in rat liver. The same effect is achieved with HICA alone whereas keto acid of leucine (α-ketoisocaproate) does not produce the same effect at normal concentrations [41]. It seems that in the present study the soccer players could benefit the supplementation of HICA. Their average protein intake was already rather high, 1.6 - 1.7 g/kg/day, and the intake of HICA per day was 1.5 g. It can be concluded that ingestion of this extra "amino acid" HICA, even with sufficient daily protein and thus probably also leucine intake, increases lean muscle mass. Probably this increase comes mainly through minimizing catabolic processes induced by exercise but needs further studies.

It must be noticed that the training period was 4 weeks which is very short time to achieve training effects. The training of the soccer players consisted of resistance training (weights) only four times during 28 days whereas 13 soccer units and three matches were included. This means that a lot of endurance (both aerobic and anaerobic) type exercises were included and probably catabolic processes in body were quite strong. For this reason HICA might have been efficient in minimizing those processes. The importance of making room for protein in muscle recovery also from endurance exercise in increasing mixed skeletal muscle fractional synthetic rate and whole body protein balance has been actively discussed recently [42,33].

### Physical performance

There were no changes in physical performance in either group during the 4-week period. This period was the last month before the competitive season and the content of the training was planned quite intensive. Consequently, it was probably too short time period to get strong training responses. Notwithstanding the increase in muscle mass of the lower extremities in HICA, full squat (1RM), 5-jump and strength endurance in squat did not improve significantly in the HICA group compared to the PLACEBO group. The reason for this may be the small number of subjects and, on the other hand, physical performance parameters improved slightly in the PLACEBO group, too.

### DOMS

The DOMS symptoms are particularly associated with the eccentric exercise [16,17]. In soccer there are a lot of unaccustomed movements (jumps in various situations) and motions (acceleration runs and braking after sprint etc.) and therefore eccentric muscle functions occur. In the present study the players marked on an average points from 1 to 3 out of 5 showing that they had all consistently some DOMS symptoms. During the last 4^th ^study week the subjects of the HICA group felt milder symptoms compared to the subjects in the PLACEBO group. Delayed presentation of the subjective effect could be explained by enzyme inhibition. We don't presently know the exact mechanism of action, but it can be speculated that decreased DOMS symptoms could be due to HICA's direct inhibitory effect on various metalloproteinase enzymes [14]. Training alertness was also increased with concomitant decrease of DOMS symptoms. That effect was significantly noted after the 2^nd ^week in the HICA group and thereafter it seemed to continue up to the last weeks.

Mixture of BCAAs has recently shown to decrease symptoms of DOMS but the most effective ratio of the three BCAAs is unclear [43]. In our pilot study with wrestlers [15; unpublished] the findings with HICA suggested that it alone is highly effective on DOMS symptoms. According to literature such effect has been described previously with the combination of α-keto isocaproic acid and β-hydroxy-β-methyl butyrate [21]. The mechanism by which HICA alleviates DOMS symptoms is unclear. Future studies are needed to compare the effects of different leucine metabolites, leucine itself and leucine-rich food in humans.

## Conclusion

HICA supplementation of 1.5 g a day leads to small increases in muscle mass during a four week intensive training period in soccer athletes.

## Competing interests

The authors Dr, MD Tuomo Karila and Dr, MD Timo Seppälä are inventors of HICA patent of "Nutrient Supplement and use of the same" and also partners at Oy Elmomed Ltd. The Study was conducted at independent research unit and the leader of the study Dr Mero and the other coauthors have no relationships to any studied substances.

## Authors' contributions

AAM conceived the study, developed the study design, participated in data acquisition and drafting the manuscript. TO developed the study design, participated in the data acquisition and assisted in drafting the manuscript. JJH assisted with the design of the study, and the manuscript preparation. RP collected blood samples and analyzed them. TS and TAMK assisted with the design of the study and drafting the manuscript. All authors have read and approved the final manuscript.
